# Paternal developmental thyrotoxicosis disrupts neonatal leptin leading to increased adiposity and altered physiology of the melanocortin system

**DOI:** 10.3389/fendo.2023.1210414

**Published:** 2023-07-25

**Authors:** Maria Elena Martinez, Zhaofei Wu, Arturo Hernandez

**Affiliations:** ^1^ Center for Molecular Medicine, MaineHealth Institute for Research, Scarborough, ME, United States; ^2^ Graduate School for Biomedical Sciences and Engineering, University of Maine, Orono, ME, United States; ^3^ Department of Medicine, Tufts University School of Medicine, Boston, MA, United States

**Keywords:** thyroid hormone, intergenerational epigenetics, adiposity, hypothalamic-pituitary-thyroid axis, fasting, leptin, energy balance, sexual dimorphism

## Abstract

**Background:**

The genetic code does not fully explain individual variability and inheritance of susceptibility to endocrine conditions, suggesting the contribution of epigenetic factors acting across generations.

**Methods:**

We used a mouse model of developmental thyrotoxicosis (*Dio3-/-* mouse) to analyze endocrine outcomes in the adult offspring of *Dio3-/-* males using standard methods for body composition, and baseline and fasting hormonal and gene expression determinations in serum and tissues of relevance to the control of energy balance.

**Results:**

Compared to controls, adult females with an exposed father (EF females) exhibited higher body weight and fat mass, but not lean mass, a phenotype that was much milder in EF males. After fasting, both EF females and males exhibited a more pronounced decrease in body weight than controls. EF females also showed markedly elevated serum leptin, increased white adipose tissue mRNA expression of leptin and mesoderm-specific transcript but decreased expression of type 2 deiodinase. EF females exhibited decreased serum ghrelin, which showed more pronounced post-fasting changes in EF females than in control females. EF female hypothalami also revealed significant decreases in the expression of pro-opiomelanocortin, agouti-related peptide, neuropeptide *Y* and melanocortin receptor 4. These markers also showed larger changes in response to fasting in EF females than in control females. Adult EF females showed no abnormalities in serum thyroid hormones, but pituitary expression of thyrotropin-releasing hormone receptor 1 and thyroid gland expression of thyroid-stimulating hormone receptor, thyroid peroxidase and iodotyrosine deiodinase were increased at baseline and showed differential regulation after fasting, with no increase in *Trhr1* expression and more pronounced reductions in *Tshr*, *Tpo* and *Iyd*. In EF males, these abnormalities were generally milder. In addition, postnatal day 14 (P14) serum leptin was markedly reduced in EF pups.

**Discussion:**

A paternal excess of thyroid hormone during development modifies the endocrine programming and energy balance in the offspring in a sexually dimorphic manner, with baseline and dynamic range alterations in the leptin-melanocortin system and thyroid gland, and consequences for adiposity phenotypes. We conclude that thyroid hormone overexposure may have important implications for the non-genetic, inherited etiology of endocrine and metabolic pathologies.

## 1 Introduction

The etiology of complex human conditions, including endocrine and metabolic disorders, is not fully understood. Despite their significant heritability, genetic factors alone cannot explain a large proportion of clinical cases (1–5), suggesting the possibility that intergenerational epigenetic effects (1, 6–8) elicited by environmental circumstances in progenitors or ancestors significantly contribute to the onset or susceptibility to these conditions. In this regard, it is increasingly appreciated that diet (9, 10), obesity (11, 12), environmental chemicals (13) and drugs (14), hormone levels (15), infection states (16), stress and trauma (17) and social interactions (18–20), among other factors, may cause changes in maternal and paternal germ line epigenetic signatures that will lead to phenotypic abnormalities in the offspring and subsequent generations, with broad implications for pathophysiology.

Despite the extensive recent literature on factors that can exert intergenerational epigenetic effects in humans and animal models, we have very limited knowledge of the roles played in this paradigm by the thyroid hormones (21). Yet the role of aberrant thyroid hormone exposure and signaling in intergenerational epigenetics is of particular importance given the high prevalence of thyroid conditions in humans (22), including pregnant women, and the ubiquitous presence in our environment of chemicals that can disrupt thyroid hormone physiology (23, 24).

Recent work has shown that thyroid hormones elicit transgenerational epigenetic effects in humans, as demonstrated by studies on multiple generation descendants of women carrying a mutation in the thyroid hormone receptor beta (15, 25). These women experience abnormally elevated thyroid hormone levels during pregnancy, and their genetically normal offspring exhibits a thyroid axis with altered sensitivity to thyroid hormones, a trait that is maintained for at least two generations across the paternal lineage (15). We have also demonstrated the occurrence of transgenerational epigenetic effects by thyroid hormones using a mouse model of genetic deficiency in the type 3 deiodinase (DIO3), the enzyme that clears both thyroid hormones (26), thyroxine (T4), and 3,5,3’-triiodothyronine (T3), which is considered the active hormone and responsible for canonical signaling due to its highest affinity for the nuclear thyroid hormone receptor (27, 28). Due to the strong developmental pattern of expression of *Dio3* (29–33), mice lacking DIO3 function exhibit pronounced fetal and neonatal thyrotoxicosis (34, 35) leading to reduced testis size (32) and decreased methylation in the neonatal testis that in adulthood translates into an aberrant sperm methylome (36). We have shown that these thyroid hormone-driven alterations in the epigenetic information of the male germ line cause abnormalities in genetically normal F2-generation descendants, affecting neonatal brain gene expression patterns and adult behavior (36).

However, the seminal work by Bakke in the 70s using rat models of adult hypothyroidism and neonatal thyrotoxicosis (thyroid hormone excess) indicated that animals subjected to these insults produced offspring with abnormal body weight and altered size of several endocrine organs (37, 38). Following on these historic and simple observations, the goal of the present study is to determine the intergenerational effects of thyroid hormones on growth and neuroendocrine physiology using the DIO3-deficiency mouse model of developmental thyrotoxicosis. Thus, here we investigate the energy balance and neuroendocrine phenotypes in the offspring of T3-overexposed males compared to that of animals of the same genotype generated by non-exposed males. Mice from T3-overexposed fathers manifest sexually dimorphic alterations in adiposity, physiological response to fasting and multiple endocrine endpoints of the leptin-melanocortin system and the thyroid axis, suggesting that thyroid hormone states in previous generations are a significant determinant of endocrine and metabolic outcomes.

## 2 Methods

### 2.1 Experimental animals and animal studies

As a model of developmental overexposure to T3, we used mice genetically deficient in the type 3 deiodinase (DIO3). We have previously described that *Dio3 -/- mice* exhibit markedly elevated serum levels of T3 during fetal and early neonatal life (34). All experimental mice were on an outbred CD-1 genetic background to overcome the impaired fertility of *Dio3-/-* mice on inbred genetic backgrounds. The original mutant mouse strain was generated in a 129/SvJ genetic background (34, 39) and has been backcrossed on a CD-1 background for more than 12 generations. Due to the genomic imprinting and preferential expression of the *Dio3* gene from the paternal allele (39–41), the colony has been maintained for more than eight generations by crossing wild type males with heterozygous females, so that the heterozygous mice generated for colony maintenance are phenotypically normal, as they carry the *Dio3* mutation in the maternal allele, which is already largely suppressed due to genomic imprinting (39, 41). To avoid the influence of confounding factors and minimize biological variability, mothers of experimental animals were mated at two months of age with 2-3 month old males. Experimental animals were born before the mother was four months old. Experimental mice represent only first litters from three to seven different mothers, and litter size was limited to 8-12 pups at birth. Mothers of experimental mice were single-caged before giving birth to avoid paternal effects and a concurrent pregnancy while raising the pups. Animals for adult studies were weaned at the age of three weeks, caged in groups of three or four, and sacrificed at 18-20 weeks of age within the third and fifth hour of the light cycle (~Zeitgeber time 3-5). All mice were maintained on a 12 h light/dark cycle, and food (Envigo-Harlan Teklad T.2918.15 for non-breeders and T.2919.10 for breeders) and water were provided *ad libitum*. For fasting experiments, mice were fasted overnight for 16 h before euthanasia. Also, some neonatal determinations were performed in mice on a 50/50 mixed 129/SvJ/C57BL/6J genetic background, using the same experimental design. This subset of experimental animals was generated by mating 129/SvJ males of the appropriate genotype with wild type (WT) C57BL/6J females. All mice were euthanized by CO_2_ asphyxiation at a rate of 30-50% volume of the euthanasia chamber per minute, according to the guidelines of the American Veterinary Medical Association. Tissues were harvested and immediately frozen on dry ice until further use. All experiments were approved by the MaineHealth Institute for Research Institutional Animal Care and Use Committee (IACUC), under current protocol number 2112.

### 2.2 Experimental design

**Figure 1:**
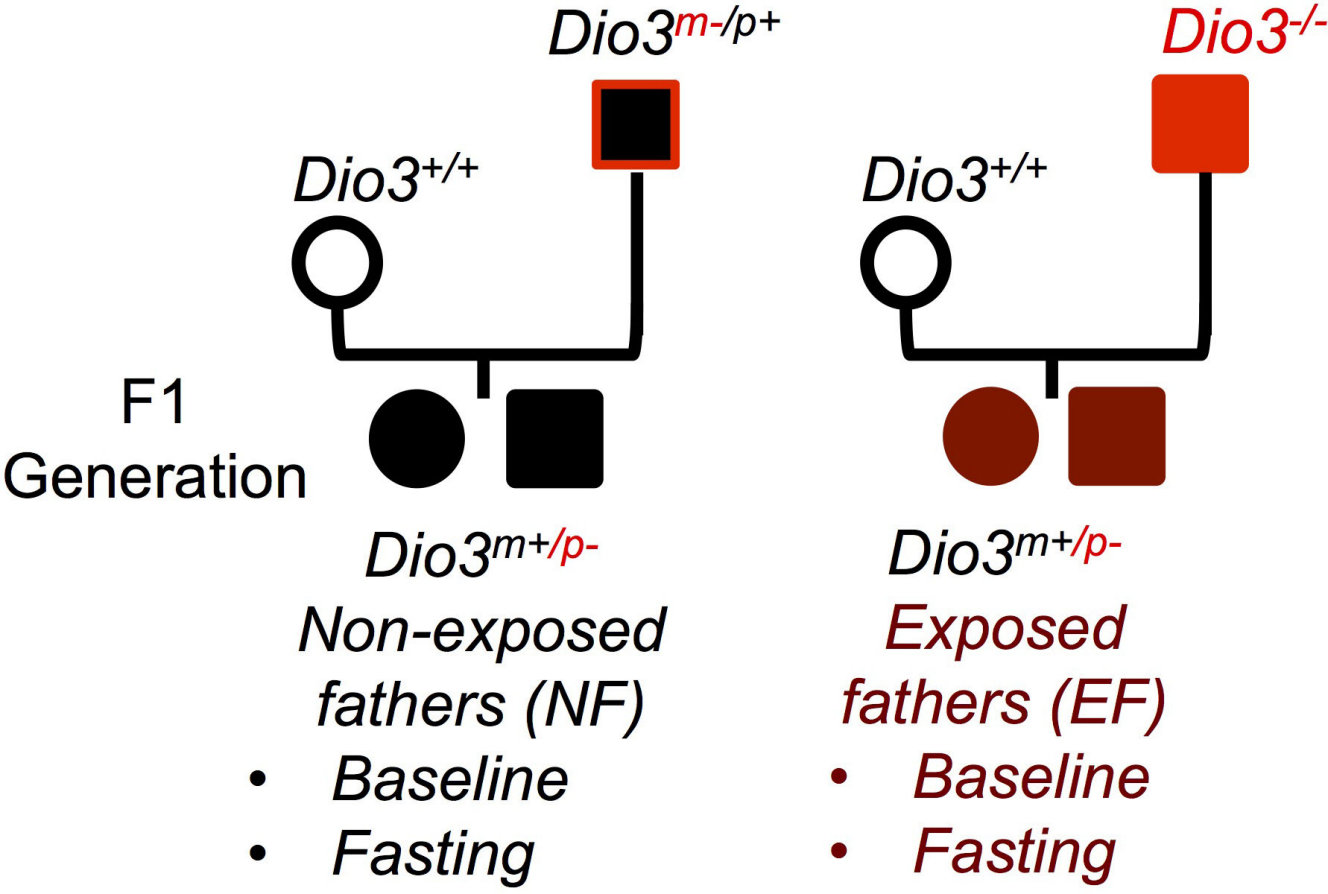
Experimental design. Mating diagrams used to generate the mice to study the net effect of paternal T3 overexposure. *Dio3 ^-/-^* mice are overexposed to T3 during fetal and early neonatal life. EF, mice generated by a T3 exposed father; NF, mice generated by non-exposed father. (Not all possible genotypes are shown).

Experimental mice were generated by crossing *Dio3-/-* males with *Dio3+/+* females (**Figure 1**). We have previously shown that *Dio3-/-* mice are overexposed to T3 during fetal and early neonatal life (34), and that the neonatal germ cells and adult sperm of *Dio3-/-* males show alterations in DNA methylation (36). The offspring of these males will be heterozygous for the *Dio3* mutation (*Dio3^m+/p-^)* and will have a mutated paternal *Dio3* allele. To generate suitable control mice, we crossed wild type females with *Dio3^m-/p+^* males (**Figure 1**). This cross will generate mice of exactly the same genotype (*Dio3^m+/p-^)* as experimental mice, but in this case the father will be phenotypically normal (not overexposed to T3 during development (40)) as the mutated allele is the maternal one. Thus, both control (non-exposed fathers, NF mice) and experimental mice (exposed fathers, EF mice) had exactly the same genotype and were born to independent wild type females, the only difference between both groups being the T3 overexposure status of the fathers.

### 2.3 Body composition and serum hormone determinations

Fat and lean mass were measured in isofluorane-anesthetized mice using a Lunar PIXImus II DEXA Densitometer (Fitchburg, WI). During the 5 min of this scanning procedure, mice were maintained anesthetized with a constant airflow containing 3% isofluorane and 3% oxygen.

Blood from adult and neonates was collected from the inferior vena cava, allowed to clot at 4 °C for at least four hours. After 10 minutes centrifugation at 3000 g, serum was taken from the supernatant and stored at -70 °C until further used. Serum levels of T3 and T4 were measured in 10 and 1 μl of serum, respectively, as previously described (42–44) by highly sensitive specific radioimmunoassays using in-house generated antibodies. Serum determinations of leptin, adiponectin and ghrelin were performed using commercially available ELISA kits according to the manufacturer’s instructions. Leptin and Adiponectin kits were purchased from R&D Systems (Bio-Techne, Minneapolis, MN, USA); ghrelin kit was purchased from EMD Millipore Corporation (Billerica, MA, USA).

### 2.4 Real time quantitative PCR

Mouse tissues were harvested and subsequently frozen on dry ice. Total RNA was extracted using the RNeasy kit from Qiagen (Valencia, CA). Total RNA (1 µg) was reverse transcribed with M-MLV reverse transcriptase in the presence of random decamers (both from Thermo Fisher Scientific, Waltham, MA) at 65°C for 5 min, then 37°C for 50 min. The 20 μl reverse transcription reactions were DNAse treated and diluted by adding 230 μl of RNase free water. An aliquot of each sample was mixed together for an internal standard and diluted fourfold. Real-time PCR reactions were set up in duplicate with gene-specific primers and SYBR Select Master Mix (Thermo Fisher Scientific, Waltham, MA), and run on the CFX Connect from Bio-Rad (Hercules, CA), where they underwent an initial 10 min denaturing step, followed by 36 cycles of a denaturing step (94°C for 30 s) and an annealing/extension step (60°C for 1 min). For each individual sample, expression was corrected by the expression of one or more (geometric mean) housekeeping genes (*Gapdh*, *Actb, Ppia or Rn18s)*, if they did not exhibit any significant difference in expression between experimental groups. Expression data are shown in arbitrary units and represented as fold-change over the mean value in the control group, or as fold-change over baseline values for fasting experiments. The sequences of the primers used for each gene determination are shown in **Supplementary Table 1**.

### 2.5 Statistical analysis

Statistical analyses were performed using the statistical tools of GraphPad Prism 6 (GraphPad Software, Inc.). Given the sexual dimorphisms in body weight and endocrine parameters, adult females and males were analyzed separately. A Student’s t-test was used to determine statistical significance at baseline between the two experimental groups. We used one-way ANOVA and Tukey’s *post hoc* test to determine statistical significance in comparisons between experimental groups or treatments. Unless otherwise indicated, responses to fasting (gene expression and hormone concentrations) were calculated as a ratio between post-fasting and corresponding baseline values, or as an increment (body composition) between post-fasting and corresponding baseline. Statistical significance was defined as P<0.05. Data are represented as the individual values of independent biological samples and the mean ± SEM. The number of mice (independent samples) used per experimental group is indicated in each figure and typically varied between 17-22 for baseline body composition, 5-12 for the rest of the adult studies, and 14-23 for neonatal studies.

## 3 Results

### 3.1 Body composition and fasting response in mice with T3-overexposed fathers

**Figure 2:**
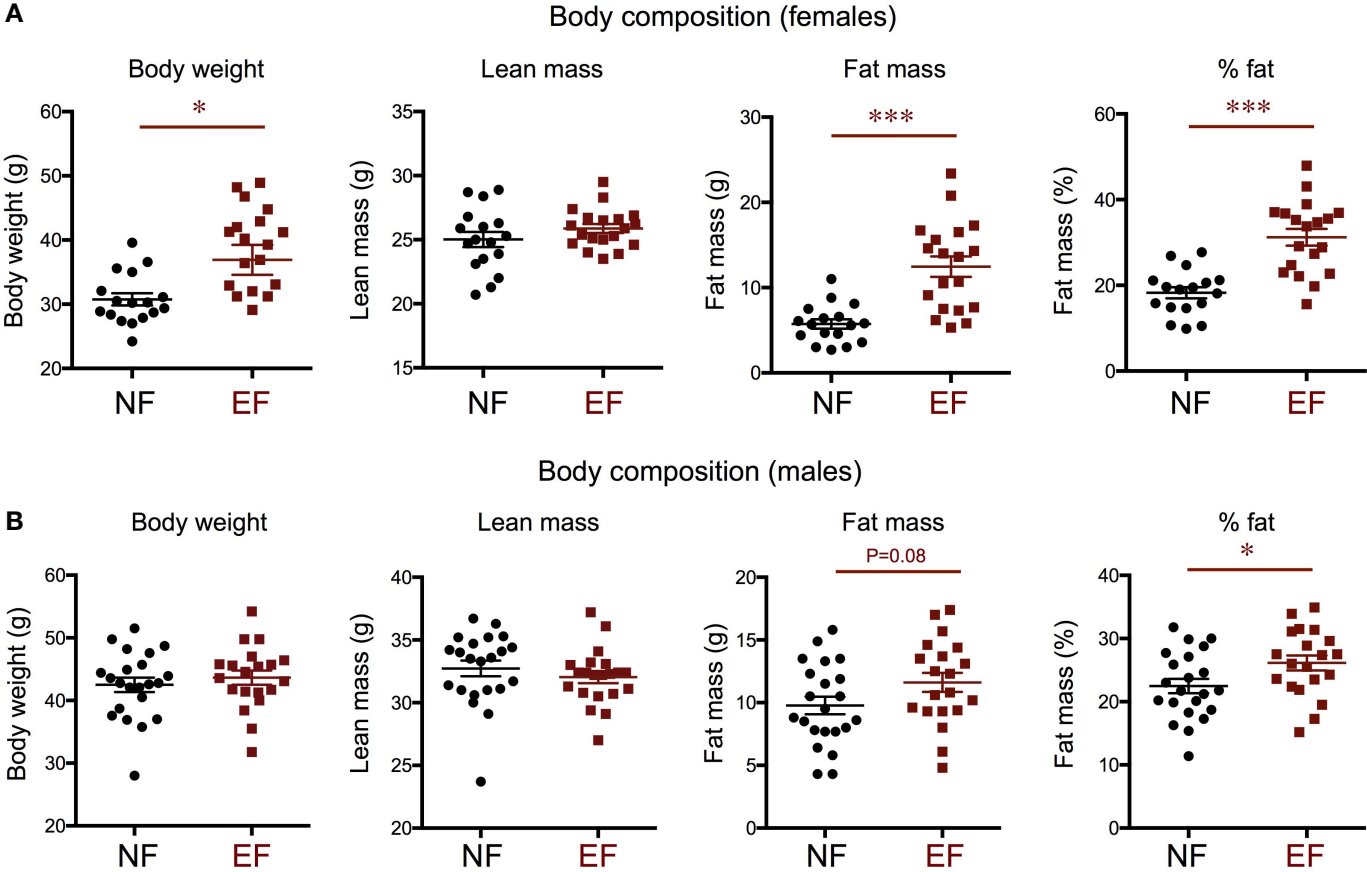
Body composition in the female **(A)** and male **(B)** offspring of T3-overexposed (EF) and non-exposed (NF) fathers. Each point represents a different animal. Experimental animals represent four to seven different litters. * and *** indicate P<0.05 and 0.001, respectively, as determined by the Student’s t-test (n=17-22).

We examined weight and body composition in mice (EF mice) fathered by males overexposed to T3 during development, and compared them with control mice (NE mice) of the same genotype (see Methods) fathered by phenotypically normal males (**Figure 1**). We observed that EF females showed a marked increase in body weight that was the result of increased adiposity and not of changes in lean mass (**Figure 2A**). Compared to control males, EF males did not exhibit significant differences in body weight or lean mass, but we noted a mild increase in fat mass and a significant increase in fat percentage (**Figure 2B**).

**Figure 3:**
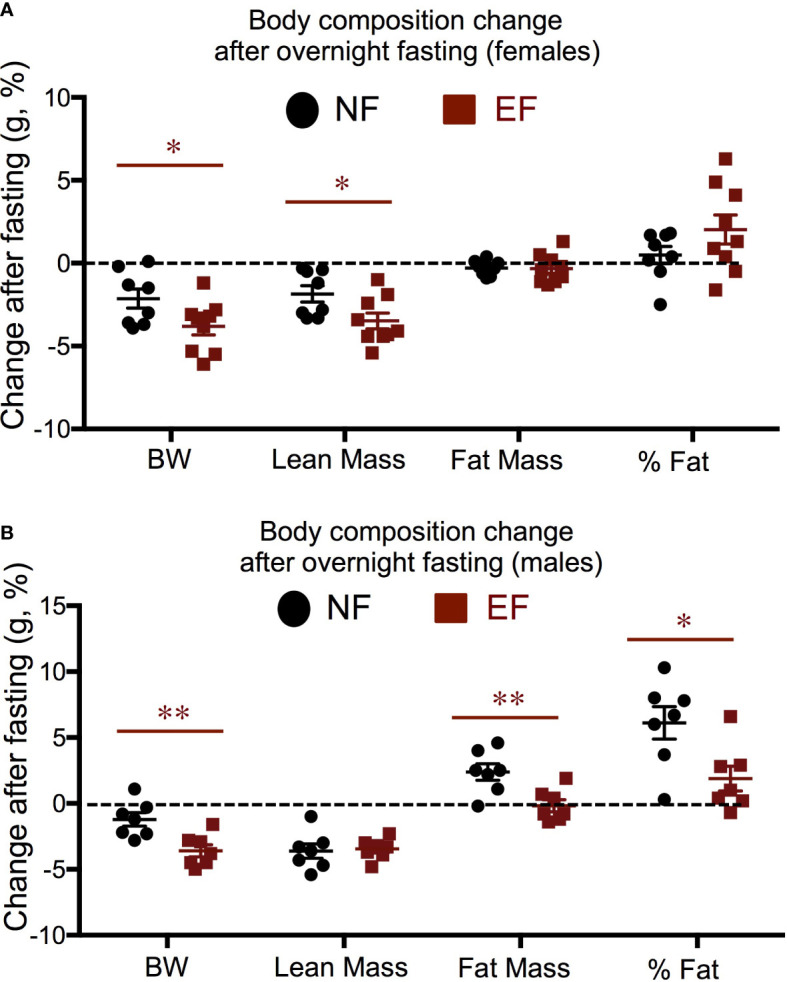
Body composition changes in response to a 16 h fasting. **(A)** Females; **(B)** Males. Data represent the % change vs baseline values. *, ** indicate P<0.05, 0.01, respectively as determined by the Student’s t-test (n=7-9).

We subjected a subset of animals to a 16 h fasting and determined individual changes in body composition. Both EF and NE female lost total weight after fasting, and this was largely due to a decrease in lean mass, not fat mass (**Figure 3A**). However, this post-fasting loss in body weight and lean mass was significantly more pronounced in EF females than in NE females (**Figure 3A**). Regarding males, both EF and NF males showed similar reductions in lean mass after fasting (**Figure 3B**), but total body weight loss was larger in EF males (**Figure 3B**). This difference, in contrast to females, was accounted for by a lack in fat mass increase, which was noted in NF males (**Figure 3B**).

### 3.2 Baseline and fasting response of thyroid axis endpoints

**Figure 4:**
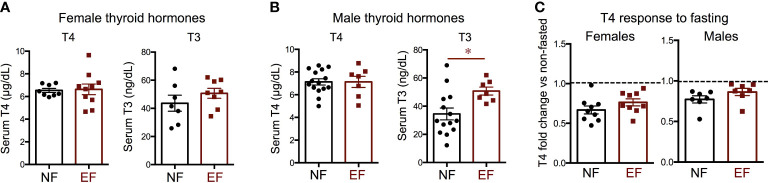
Baseline thyroid hormones and T4 change after fasting. Serum thyroid hormone levels in females **(A)** and males **(B)**; **(C)** Change in T4 after fasting in females and males. * indicate P<0.05 as determined by the Student’s t-test (n=7-15).

We then investigated hormone levels and gene expression of relevance to the thyroid axis. We observed no differences between EF and NF females in serum levels of T3 and T4 (**Figure 4A**). In males, no difference in serum T4 was noted, but EF males showed significantly increased serum T3 (**Figure 4B**). After fasting, females and males of both experimental groups manifested a reduction of serum T4 from baseline levels, but this reduction was comparable between EF and NF mice of either sex (**Figure 4C**).

**Figure 5:**
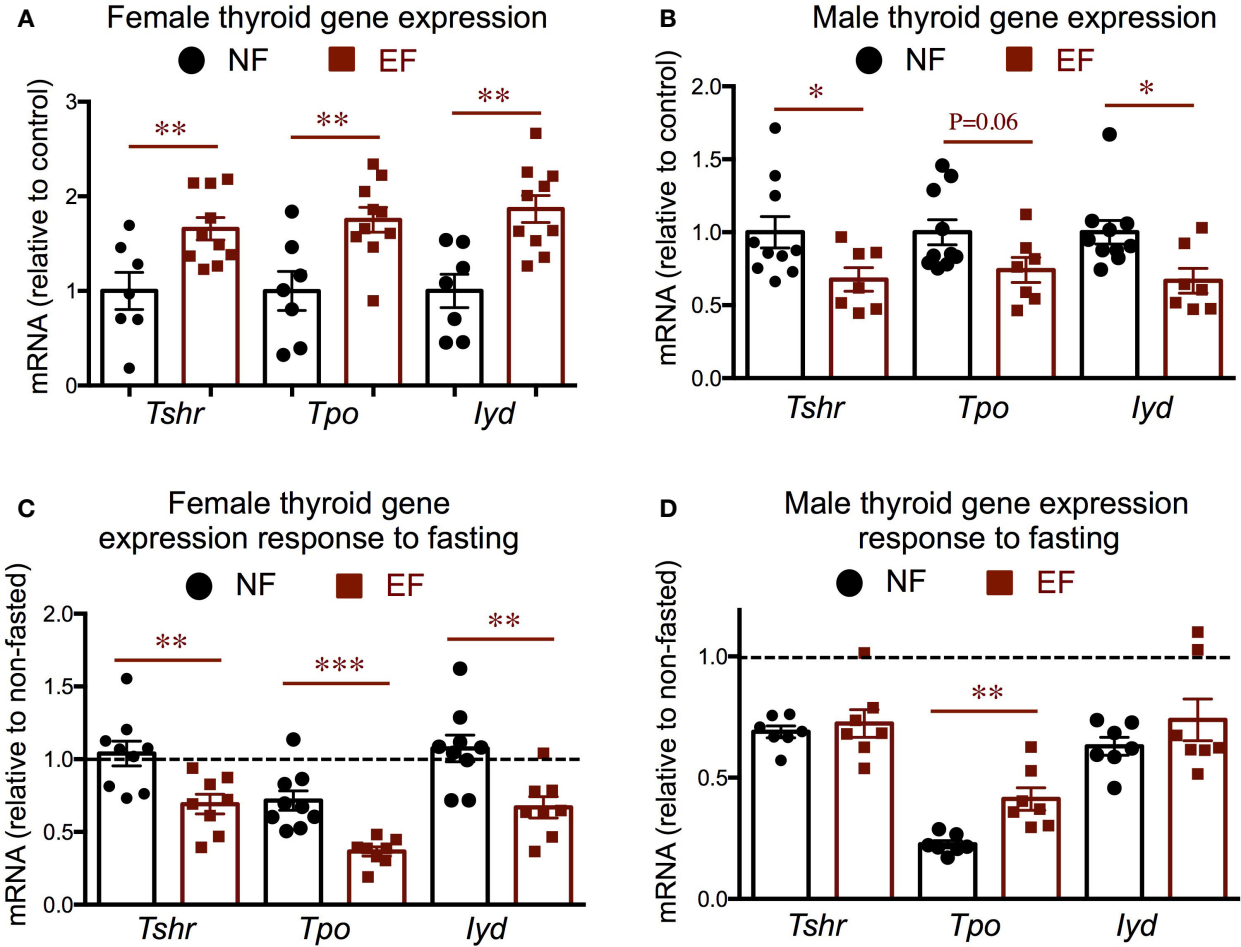
Baseline and post-fasting thyroid gland gene expression. **(A, B)** Baseline gene expression in females **(A)** and males **(B)**; **(C, D)** Post-fasting fold change in gene expression over corresponding baseline values in females **(C)** and males **(D)**. *, **, *** indicate P<0.05, 0.01 and 0.001, respectively as determined by the Student’s t-test **(A, B)** or by one-way ANOVA and Tukey’s *post hoc* test **(C, D)** (n=7-10).

We measured gene expression in the thyroid gland of markers thyroid-stimulating hormone receptor (*Thsr)*, thyroid peroxidase *(Tpo)* and iodotyrosine deiodinase (*Iyd*). We observed that the expression of all three markers at baseline was significantly elevated in EF females (**Figure 5A**), but significantly decreased in EF males (**Figure 5B**). Although the expression of these markers was higher in EF female thyroid glands, their expression after fasting was more pronouncedly reduced from baseline levels than in NF females. (**Figure 5C**). In contrast, fasting elicited comparable reductions in the expression of these markers in EF and NF males, and we only noted that *Tpo* fasting response was not as large in EF males as in NF males (**Figure 5D**).

**Figure 6:**
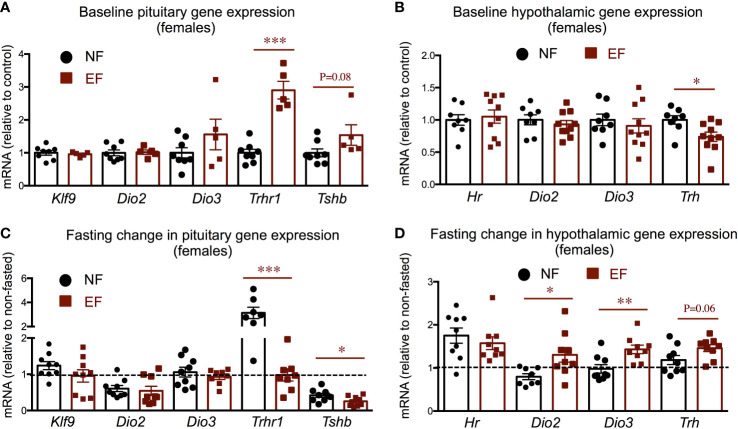
Female baseline and post-fasting pituitary and hypothalamic expression of thyroid axis genes. **(A, B)** Baseline gene expression in the pituitary **(A)** and hypothalamus **(B)**. **(C, D)** Post-fasting change in gene expression in the pituitary **(C)** and hypothalamus **(D)**. *, **, *** indicate P<0.05, 0.01 and 0.001, respectively as determined by the Student’s t-test **(A, B)** or by one-way ANOVA and Tukey’s *post hoc* test **(C, D)** (n=7-9).

Pituitary and hypothalamic gene expression did not show major differences between EF and NF females. We observed increased pituitary *Trhr1*expression in EF females, and a slight, not significant, increased in *Tshb* expression but no changes in type 2 deiodinase *(Dio2), Dio3*, or T3 target kruppel-like factor 9 (*Klf9)* (**Figure 6A**). EF females also exhibited decreased hypothalamic expression of thyrptropin-releasing hormone (*Trh)* at baseline, but no differences in the expression of *Dio2, Dio3* or T3-target hairless (*Hr)* (**Figure 6B**). We observed no differences between EF and NF females in the fasting response of pituitary expression of *Klf9, Dio2* or *Dio3*. However, pituitary thyrotropin-releasing hormone receptor 1 (*Trhr1*) expression robustly increased in NF females after fasting, but this response was not observed in EF females (**Figure 6C**). In addition, a post-fasting reduction in pituitary thyroid-stimulating hormone beta chain (*Tshb)* expression was noted in both female groups, but this response was significantly more pronounced in EF females (**Figure 6C**). Regarding hypothalamic expression, we observed a differential response to fasting in the expression of *Dio2* and *Dio3*, while the increased expression of T3-target *Hr* was similar in both groups (**Figure 6D**).

In males, thyroid axis-related pituitary and hypothalamic expression did not show remarkable differences between EF and NE groups (**Supplementary Figure 1A–D**). At baseline, pituitary expression of *Dio3* was reduced in EF males, a trend also observed in *Trhr1* expression (**Supplementary Figure 1A**), while experimental groups showed no differences in the hypothalamic expression of the genes examined (**Supplementary Figure 1B**). After fasting, male pituitary expression of *Dio2, Dio3, Trhr1* and *Tshb* was decreased to different extents compared to baseline levels, but these reductions were similar between EF and NF males (**Supplementary Figure 1C**). In the hypothalami of males, fasting elicited minor or no changes in gene expression, and no differential responses were observed between EF and NF males (**Supplementary Figure 1D**).

### 3.3 Baseline and fasting response of leptin-melanocortin system and energy balance endpoints

**Figure 7:**
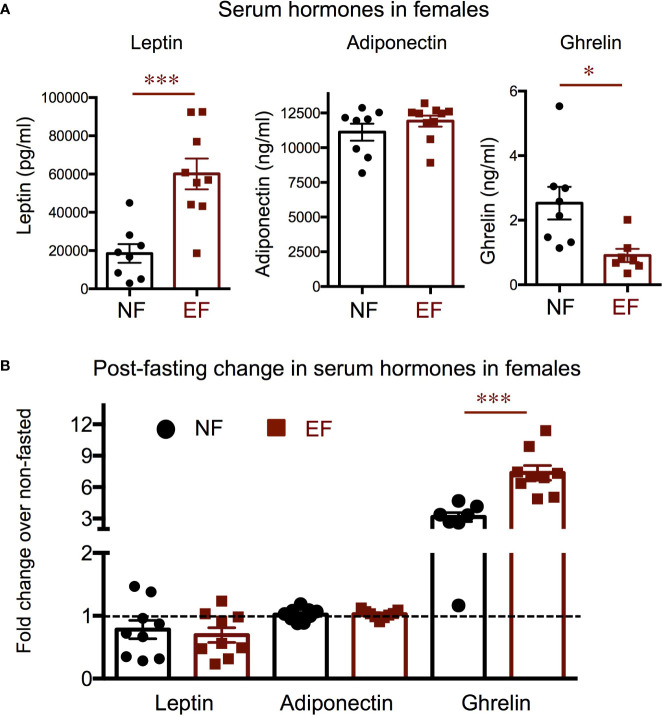
Female baseline and post-fasting serum levels of hormones regulating energy balance. **(A)** Baseline serum hormone levels. **(B)** Fold change in hormone levels in response to 16 h fasting. *, *** indicate P<0.05, 0.001, respectively, as determined by the Student’s t-test **(A)** or by one-way ANOVA and Tukey’s *post hoc* test **(B)** (n=7-9).

We also investigated hormone and molecular endpoints of the leptin-melanocortin system that may be associated with the adiposity phenotypes. Consistent with their body composition, serum leptin was markedly elevated in EF females, while we observed no differences in serum adiponectin (**Figure 7A**). After fasting, leptin levels were slightly reduced in both experimental groups of females, but the response was comparable in both experimental groups (**Figure 7B**). Serum adiponectin did not change in either group after fasting (**Figure 7B**). Interestingly, we observed that serum ghrelin, an orexigenic hormone, was significantly reduced at baseline in EF females (**Figure 7A**). After fasting, as expected, serum ghrelin was markedly increased in both experimental groups, but the increase was significantly larger in EF females (**Figure 7B**).

We observed no differences between EF and NF males in serum levels of leptin, adiponectin or ghrelin (**Supplementary Figure 2A**). However, the post-fasting decrease in serum leptin was significantly more pronounced in EF males than in NF males (**Supplementary Figure 2B**). Fasting adiponectin in EF males also trended lower than in NF males, although it did not reach statistical significance (**Supplementary Figure 2A**). The changes in serum ghrelin after fasting were large in both groups of males and of similar magnitude.

**Figure 8:**
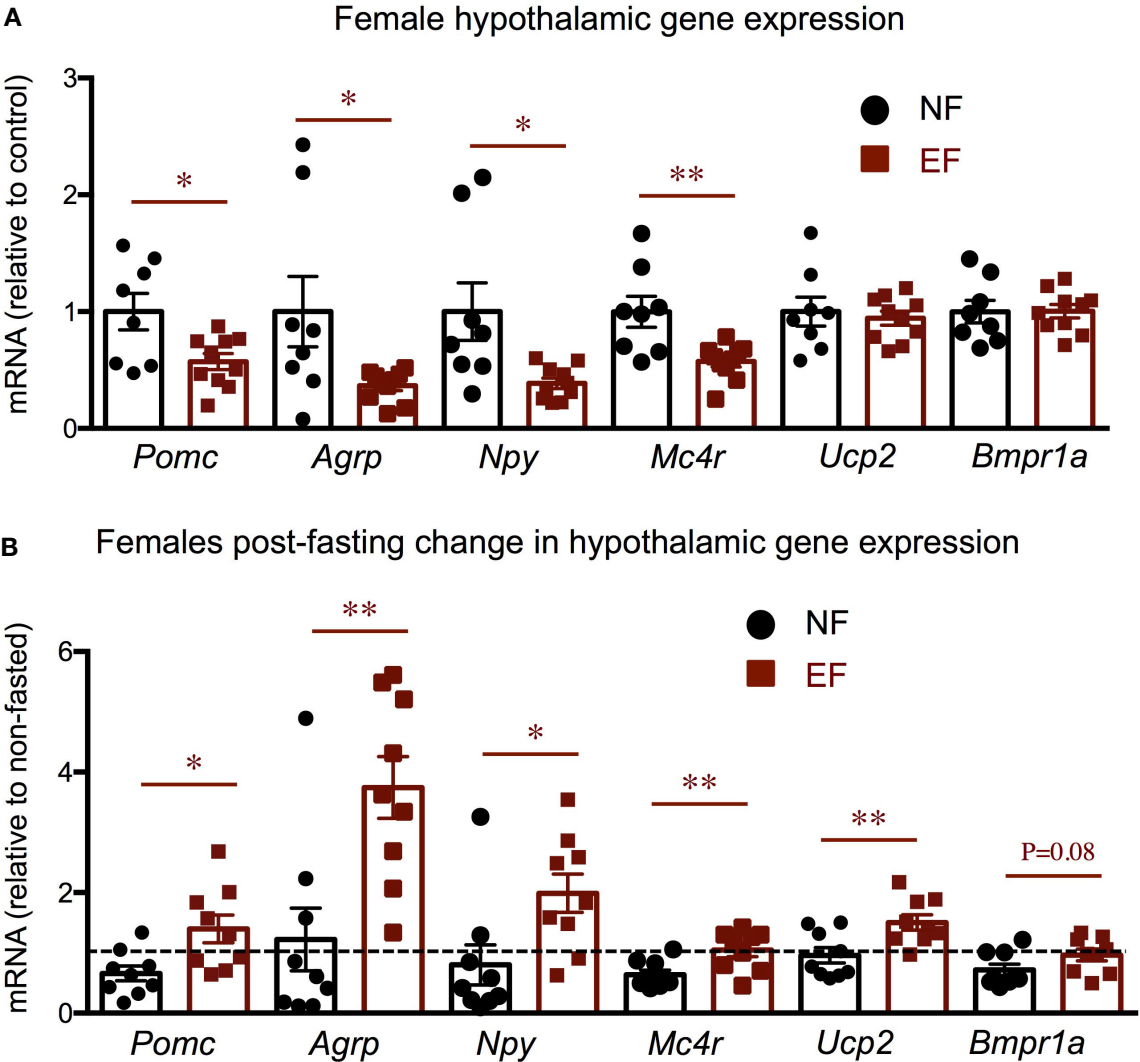
Female baseline and post-fasting hypothalamic expression of energy balance related genes. Baseline **(A)** and post-fasting change **(B)** in hypothalamic gene expression. * and ** indicate P<0.05 and 0.01, respectively, as determined by the Student’s t-test **(A)** or by one-way ANOVA and Tukey’s *post hoc* test **(B)** (n=8-10).

We then determined the hypothalamic expression of genes related to the leptin-meanocortin system and energy balance. We observed that EF females show significant decreases in the hypothalamic expression of leptin-melanocortin system components, including pro-opiomelanocortin (*Pomc)*, agouti-related peptide *(Agrp)*, neuropeptide Y *(Npy)* and melanocortin receptor 4 (*Mc4r)* (**Figure 8A**), while no differences were noted in the expression of other determinants of energy balance like uncoupling protein 2 (*Ucp2)* and bone morphogenetic protein receptor 1a (*Bmpr1a)*. After overnight fasting, responses relative to their corresponding baselines were significantly more pronounced in EF females, with larger changes in all the markers measured (**Figure 8B**).

These results were sexually dimorphic, as they were not observed in EF males. At baseline, hypothalamic expression of all those markers was not different between EF and NF males (**Supplementary Figure 3A**). In this regard, fasting responses in EF and NF males were similar for *Pomc, Agrp, Npy* and *Ucp2*, while we observed a modest difference for *Mc4r* and *Bmpr1a*. For these genes, the post-fasting expression levels were slightly different between experimental groups, with their expression values in EF males being higher than in NF males due to an absence of post-fasting decrease or the presence of a post-fasting increase, respectively (**Supplementary Figure 3B**).

**Figure 9:**
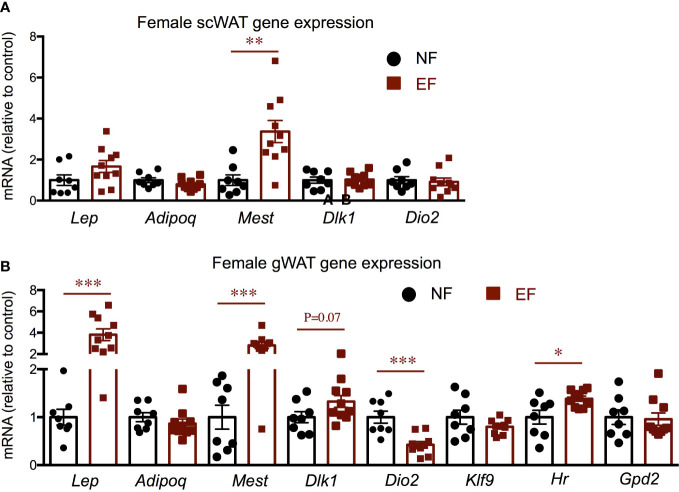
Female gene expression in WAT depots. Gene expression in subcutaneous WAT **(A)** and in gonadal WAT **(B)**. *, **, *** indicate P<0.05, 0.01 and 0.001, respectively as determined by the Student’s t-test (n=8-10).

Given the increased adiposity in EF females, we explored relevant gene expression in white adipose tissue (WAT). Compared to control females, the sub-cutaneous WAT of EF females showed increased expression of adiposity markers mesoderm-specific transcript *(Mest)* and leptin (*Lep*) (the latter did not reach statistical significance), but no differences in the expression of adiponectin (*Adipoq*), adipocyte progenitor marker delta-like non canonical notch ligand 1 (*Dlk1)* or T3-generating, beiging marker *Dio2* (**Figure 9A**). However, gonadal WAT manifested remarkable changes in gene expression. In this fat depot, adiposity markers *Mest* and *Lep* were strongly up-regulated, while *Dlk1* expression trended higher (**Figure 9B**). Importantly, we also observed that *Dio2* expression was markedly suppressed in EF gonadal fat, but this decrease was not accompanied by reduced expression of T3 target genes *Klf9, Hr* and glycerol phosphate dehydrogenase 2 (*Gpd2)* (**Figure 9B**).

### 3.4 Neonatal leptin and T4

**Figure 10:**
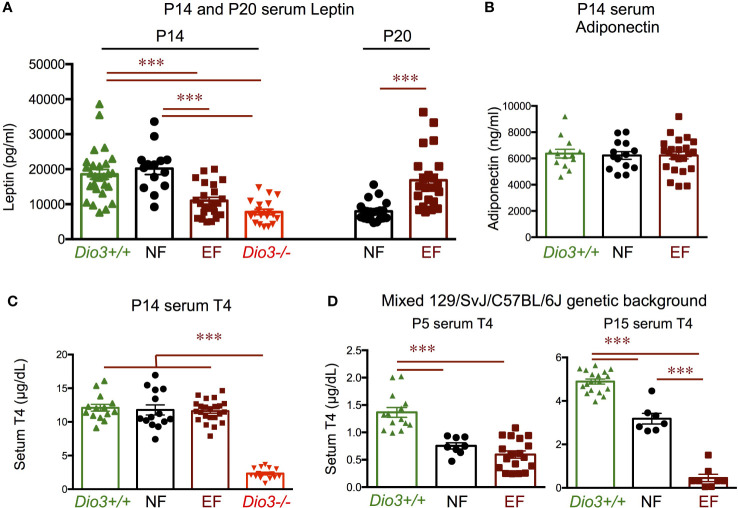
Postnatal hormone profiles. **(A)** Seum leptin in P14 and P20 mice of both sexes. **(B)** Serum adiponectin in P14 mice of both sexes. **(C)** Serum T4 in P14 mice of both sexes. **(D)** Serum T4 in P5 and P15 mice of both sexes on a mixed 129/SvJ/C57BL/6J genetic background. *** indicate P< 0.001 as determined by the Student’s t-test (P20 data) or by one-way ANOVA and Tukey’s *post hoc* test (P5, P14 and P15 data) (n=8-27).

We then examine serum levels of leptin and T4 during late neonatal development. At postnatal day 14 (P14), serum leptin was markedly reduced in EF mice compared to NF mice (**Figure 10A**). The lower leptin in EF mice was comparable to that in their T3-overexposed fathers (*Dio3-/-* males) at the same age, while the higher leptin levels in NF mice were similar to those in genetically intact *Dio3+/+* mice (**Figure 10A**). At P20, serum levels were significantly higher in EF mice than in NF mice (**Figure 10A**), suggesting a pronounced disruption in serum leptin ontogeny in EF animals. At P14, we observed no differences in serum adiponectin between EF and NF mice, and values were similar to those in *Dio3+/+* animals (**Figure 10B**). Similarly, EF, NF and *Dio3+/+* mice showed comparable levels of serum T4 at P14, while values at this age for *Dio3+/+* mice were markedly suppressed (**Figure 10C**).

Interestingly, using the same experimental design with animals on a 50/50 mixed genetic background (129/SvJ with C57BL/6J, see Methods), we observed that both EF and NF mice have reduced serum T4 compared to *Dio3+/+* mice at P4 (**Figure 10D**). By P15, we still observed lower serum T4 in EF and NF mice compared to *Dio3+/+* values, but there was a pronounced divergence in serum T4 between EF and NF mice, with the latter trending higher and closer to *Dio3+/+* values, while T4 in EF mice remained strongly suppressed, with values comparable to those in *Dio3-/-* mice (**Figure 10D**).

## 4 Discussion

Mice with global DIO3 deficiency provide a robust, consistent model of fetal and early neonatal thyrotoxicosis (34, 40). Using this model, we have recently shown that developmental T3 excess modifies the epigenetic information of the male germ line (36), with potential consequences for phenotypic alterations in the next generation (36, 45). This possibility was supported by seminal work by Bakke using a model of neonatal injections of pharmacological doses of T4 (neo-T4 model) (37, 46). Given that DIO3-deficient male (and female) mice exhibit marked alterations in adiposity and in the physiology of the thyroid axis and the leptin-melanocortin system, we investigated those phenotypes in the offspring of DIO3-deficient males.

In contrast to their fathers, which show reduced growth and decreased adiposity (47), the offspring of *Dio3-/-* males exhibited an increase in body weight in females, but not in males. This observation is consistent with old studies using neo-T4 rats as a model of neonatal thyrotoxicosis, and showing that the female –but not male- offspring of neo-T4 males also exhibited elevated body weight (37). Although that previous study did not provide information about fat mass, in our study, the increased body weight in EF females is largely the result of increased adiposity. Not surprisingly, the obesity phenotype in EF females was associated with a higher level of serum leptin, and with increased WAT expression of *Lep* and *Mest*, two well-established markers of adipose tissue expansion (48–51). Furthermore, EF female WAT also showed reduced expression of *Dio2*, an enzyme activated by adrenergic stimuli (52) and that participates in the “beiging” of adipose tissue (53, 54) by locally generating T3 and increasing T3 action (55). Thus the reduction of *Dio2* suggests a lower metabolic level in the WAT of these females. The more pronounced abnormal gene expression profile in gonadal WAT compared to subcutaneous WAT is consistent with observations of differential physiology of different WAT depots (56–58).

The obesity phenotype of EF females suggested abnormalities in energy balance that might be a consequence of altered programming of the leptin-melanocortin system, as previously shown for their *Dio3-/-* fathers (47). Indeed, the decreased baseline expression of hypothalamic leptin-dependent markers *Pomc, Agrp, Npy* and *Mc4r*, as well as their enhanced response to a fasting challenge, indicate an abnormal set-point and a larger dynamic range in the leptin-melanocortin system of EF females, which could be the cause of an abnormal energy balance and obesity. Interestingly, EF females show at baseline lower serum level of ghrelin, an orexigenic peptide (59), but the change in ghrelin after wasting was much larger, again suggesting an abnormally expanded range in physiological mechanisms affecting feeding.

EF females also exhibited differences in body composition changes after overnight fasting. Although both groups of females lost body weight, EF females lost more weight than controls, and this was largely explained by a more pronounced reduction in lean mass, while fat mass remained at similar levels. As the leptin-melanocortin system has critical role in the physiological response to fasting (60), the differential sensitivity to fasting in body composition further illustrates the abnormal programming of this system in EF females.

Thyroid hormones are also critical regulators of metabolism and energy balance (61), and the initial observations by Bakke et al. suggested alterations of the hypothalamic-pituitary-thyroid axis in the offspring of Neo-T4 males (37). Although we did not observed differences between experimental female groups in serum levels of thyroid hormones at baseline or after fasting, the expression of thyroid markers in EF females thyroid glands was significant increased, an observation that could be consistent with thyroid gland hypertrophy, and with the thyroid gland enlargement previously reported in the female offspring of Neo-T4 males (37). Although the elevation in pituitary *Tshb* expression in EF females did not reach statistical significance, it was also observed in the female offspring of neo-T4 males (37), and it could explain the increased expression of thyroid markers as a result of elevated TSH action on the thyroid gland. Still the marked difference in the fasting response of pituitary *Tshb* expression suggests an abnormal physiological programming. Thus, although unchanged thyroid hormone concentrations do not explain the adiposity phenotype, the serum, thyroid gland and pituitary endpoints in EF females all point to an abnormally regulated HPT axis. Although only paraventricular nucleus TRH is hypophysiotropic, the reduced hypothalamic expression of TRH but modest increase in pituitary expression of *Tshb* also suggests altered pituitary sensitivity to TRH, a phenotype that has also been observed in relevant rat (37) and human (15) models of paternal thyrotoxicosis.

The abnormal programming of endocrine systems in the offspring of males that experienced T3 excess during development is likely the result of altered epigenetic information in the sperm of these males (36), leading to disrupted development in the offspring. In this regard, aberrant ontogeny of leptin and thyroid hormones may play a role in the adult phenotypes observed. Serum leptin exhibits a marked peak around P10 in mice (62), and a large body of literature indicates that this peak exerts important trophic effects in the maturing hypothalamus. Alterations in the neonatal peak of leptin have demonstrated its importance not only for the programming of the hypothalamic melanocortin system and associated feeding and energy balance circuitries (63–73), but also for the physiology of other hypothalamic-pituitary neuroendocrine systems (74–76). In this context, the markedly reduced serum leptin manifested by P14 EF mice of both sexes could be indicative of an abnormal leptin profile during neonatal life, and possibly of a blunted leptin peak, which could partially explain the hypothalamic-related phenotypes in adult animals. However, our data represent only one time point and P14, chosen for these initial studies due to thyroid hormones peaking at this time, is not the ideal timing for measuring peak leptin. Thus, demonstration that neonatal leptin abnormalities are indeed causing the adult endocrine phenotypes of EF mice will require more comprehensive studies of neonatal leptin profiles and possible experiments of phenotype rescue by timely neonatal administration of leptin. In this regard, changes in neonatal leptin profiles have also been previously associated with the intergenational effects of maternal nutrition status in a sheep model (77). Interestingly, the reduced neonatal leptin in EF mice is comparable to that in their *Dio3-/-* fathers, suggesting a paternal epigenetic transmission of neonatal leptin phenotypes.

Developmental alterations in serum thyroid hormones could also be contributing to the adult phenotypes of EF mice. For example, neonatal excess in T3 levels, as that observed in *Dio3-/-* mice and neo-T4 rats, also influences the programming of HPT axis and leptin-melanocortin system (47, 78, 79). Low neonatal levels of thyroid hormone can also determine hypothalamic leptin signaling in adult life (80). However, we observed no alterations in that regard. P14 levels of T4 in EF mice were the same as in the NF control group and in *Dio3+/+* mice, even though we observed a marked excess-T3 suppresion of serum T4 in their *Dio3-/-* fathers, consistent with previous reports (34). It is still possible that alterations in serum thyroid hormones occur in the EF mice studied at earlier ages and are then normalized by P14. This possibility is illustrated by the results we obtained from mice on a different genetic background. On a mixed C57BL/6J/129/SvJ genetic background serum T4 in P4 EF mice is strongly suppressed to a comparable degree to that in *Dio3-/-* mice. At this age NF mice also exhibit a milder T4 suppresion. T4 remains suppressed by P14 in EF mice and partially normalizes in NF mice, suggesting a pattern of normalization with late neonatal development, and opening the possibility that the adult phenotypes identified in our main experiment are also partly the result of aberrant thyroid hormone levels in early life. These results also suggest the severity of some T3-induced intergenerational epigenetic effects is dependent on the genetic background of the animals, a notion that has been noted in other paradigms of intergenerational effects (81).

Although the phenotypes observed in EF males are milder than those in females, their percentage of body fat, body composition response to fasting, thyroid gland markers and post-fasting leptin, to name a few parameters, are still significantly abnormal, also suggesting an endocrine programming different from controls that could potentially affect other physiological systems, or be more overtly revealed by other types of physiological challenges. Considering the likely developmental origins of the phenotypes observed, as discussed above, the sexual dimorphism in adult outcomes may be the result of leptin and thyroid hormone cross-talk with mechanisms of hypothalamic sexual differentiation during neonatal life.

In summary, our results demonstrate that paternal alterations in thyroid hormone states may cause broad effects in the neuroendocrine physiology of the offspring, affecting their susceptibility to obesity and endocrine disease. These outcomes likely develop from aberrant neonatal leptin or other hormones as a result of the altered epigenetic information that is transmitted *via* the sperm of fathers overexposed to T3. In humans, this paradigm may have important implications for susceptibility to endocrine disease in children whose fathers were exposed to excessive T3 signaling while *in utero* due to maternal thyroid disease.

## Data availability statement

The original contributions presented in the study are included in the article/**Supplementary Material**. Further inquiries can be directed to the corresponding author.

## Ethics statement

The animal study was reviewed and approved by MaineHealth Institute for Research Instituional Animal Care and Use Committee.

## Author contributions

MEM and ZW performed all the experiments, generated and analyzed data, and drafted results and figures. AH designed the study and wrote the manuscript. All authors contributed to the article and approved the submitted version.
