# Supplementary material for: Paternal developmental thyrotoxicosis disrupts neonatal leptin leading to increased adiposity and altered physiology of the melanocortin system
Source: Front Endocrinol (Lausanne). 2023 Jul 25;14:1210414. doi: 10.3389/fendo.2023.1210414 (PMC10407661; doi:10.3389/fendo.2023.1210414)
Supplement: Supplementary file 1 [file DataSheet_1.pdf]

**Supplementary Table 1**	**Forward Primer**	**Reverse Primer**
Gene name	*Primer sequence 5'-3'*	*Primer sequence 5'-3'*
Actb	TGGGTATGGAATCCTGTGGC	CTGCATCCTGTCAGCAATGC
Adipoq	GGAATGACAGGAGCTGAAGGGC	ACAGTGACGCGGGTCTCCAGC
Agrp	GCAGACCGAGCAGAAGAAGT	ATTGAAGAAGCGGCAGTAGC
Bmpr1a	GTGTGTGAAACGCTTGCGGCCAATC	GTGAGTCTGGAGGCTGGATTATGGG
Dio2	CCTCCTAGATGCCTACAAACAGG	CATTCGGCCCCATCAGCGGTC
Dio3	TGAGCACAGCCACAGAACTC	AAAGCTGTCAGTTCGAGCCA
Dlk1	GCTGGGACGGGAAATTCTG	CCTGGCCCTCATCATCCAC
Gapdh	AGGAGCGAGACCCCACTAAC	CGGAGATGATGACCCTTTTG
Gpd2	CACTAGATGCCGTCACCAGAG	GAAGGGCTTCTTTCACCATCC
Hr	AACCCTGCATCCAAGTAGCA	AGCACTGTGTGGCA GTGTT
Iyd	CCCTGGGTGGATGAAGACTTG	GGGTATCGGGTGTGAGAGAAC
Klf9	AAGGGCCGTTCACCTGTATG	GGCTGTGGGAAAGTCTATGG
Lep	GGAGACCCCTGTGTCGGTTCCT	GCGGATACCGACTGCGTGTGTG
Mc4r	CCGGACGGAGGATGCTATGAGCA	GGCTATCGCCACGATCACTAGAATG
Mest	ATGACGGCAACCTGGTCATC	CAGAATCGACACTGTGGACC
Npy	TACTACTCCGCTCTGCGACA	TCACCACATGGAAGGGTCTT
Pomc	CACCACGGAGAGCAACCT	GTTTTCAGTCAGGGGCTGTT
Ppia	CCGTGTTCTTCGACATCACG	CAGTGCTCAGAGCTCGAAAG
Rn18	GGAGTATGGTTGCAAAGCTG	TCGCTCCACCAACTAAGAAC
Tpo	GACCATGCAGTCTACAACACC	GGACTCGGGCAGCTTAAAGAA
Trh	GGCTCAGCATCTTGGAAAGCTCTGC	CAAGGCGCAGGATTTGGGGATACCA
Trhr1	CCTTCACTGTGGAGAGGTAC	AAGAGTCCTGTAGGGCATCC
Tshb	CAACACCACCATCTGTGCTG	CAGACATCCTGAGAGAGTGC
Tshr	CACCAGGAGGACTTCAGA	GGCAGACTCGAAAATGCAAGA
Ucp2	CGAGGGGATCGGGCCATGGTA	GATTTCCTGCTACCTCCCAGAAGATGG

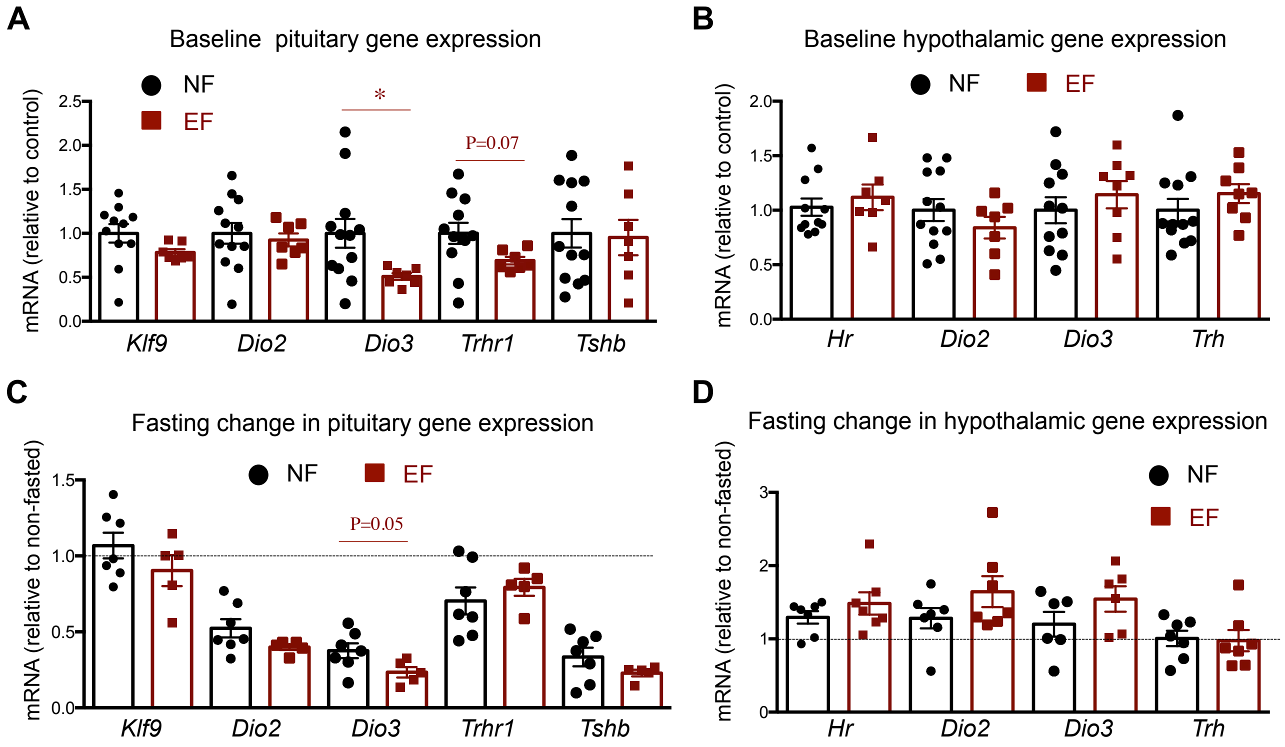

Supplementary Figure 1 Male baseline and post-fasting pituitary and hypothalamic expression of thyroid axis genes. (**A** and **B**), Baseline gene expression in the pituitary (**A**) and hypothalamus (**B**). (**C** and **D**) Post-fasting change in gene expression in the pituitary (**C**) and hypothalamus (**D**). * indicate P<0.05 as determined by the Student's t-test (A, B) of by one-way ANOVA and Tukey's post hoc test (C, D).

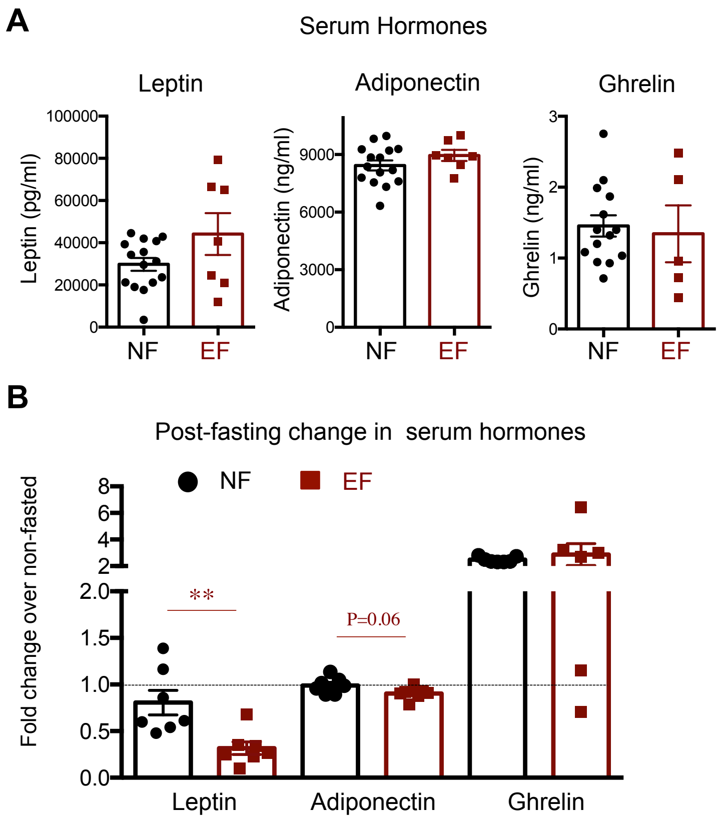

Supplementary Figure 2 Male baseline and post-fasting serum levels of hormones regulating energy balance. (**A**) Baseline serum hormone levels. (**B**) Fold change in hormone levels in response to 16 h fasting . ** indicate P< 0.01 as determined by the Student's t-test (A) of by one-way ANOVA and Tukey's post hoc test (B).

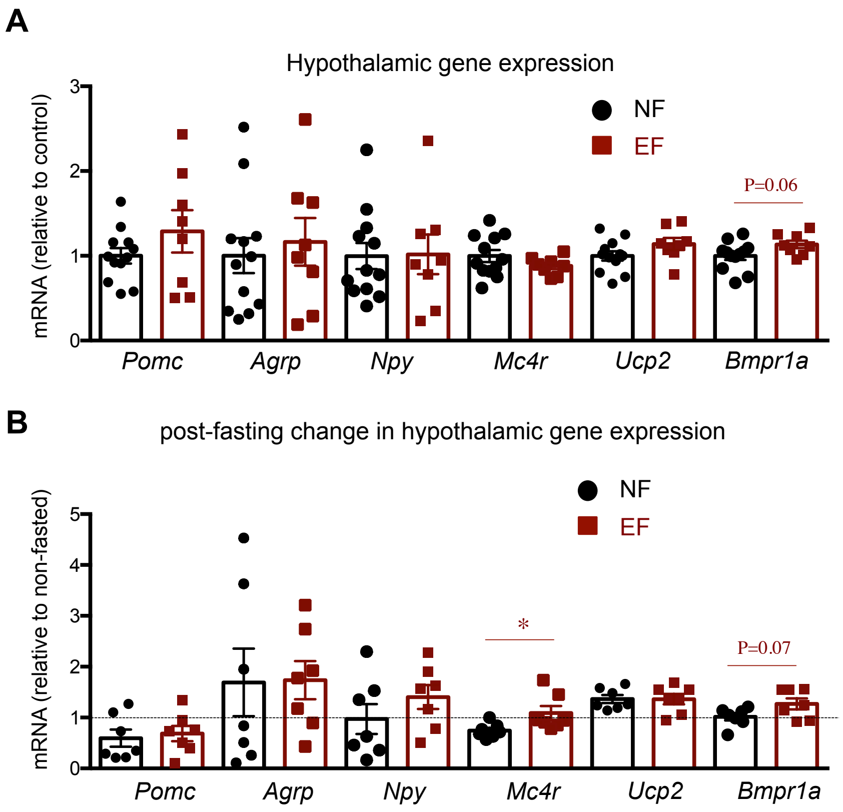

Supplementary Figure 3 Male baseline and post-fasting hypothalamic expression of energy balance related genes**.** (**A**) and post-fasting change (**B**) in hypothalamic gene expression. * indicates $P<0.05$ as determined by one-way ANOVA and Tukey's post hoc test (B).
